# A methodology for mapping current and future heat stress risk in pigs

**DOI:** 10.1017/S1751731120000865

**Published:** 2020-09

**Authors:** J. Y. Mutua, K. Marshall, B. K. Paul, A. M. O. Notenbaert

**Affiliations:** 1 Tropical Forages Program, International Center for Tropical Agriculture (CIAT), Duduville Campus, Off Kasarani Road, PO Box 823-00621, Nairobi, Kenya; 2 Livestock Genetics Program, International Livestock Research Institute (ILRI), Old Naivasha Road, PO Box 30709-00100, Nairobi, Kenya

**Keywords:** climate change, livestock, regional environmental change, climate change adaptation, spatial analysis

## Abstract

Heat stress is a global issue constraining pig productivity, and it is likely to intensify under future climate change. Technological advances in earth observation have made tools available that enable identification and mapping livestock species that are at risk of exposure to heat stress due to climate change. Here, we present a methodology to map the current and likely future heat stress risk in pigs using R software by combining the effects of temperature and relative humidity. We applied the method to growing-finishing pigs in Uganda. We mapped monthly heat stress risk and quantified the number of pigs exposed to heat stress using 18 global circulation models and projected impacts in the 2050s. Results show that more than 800 000 pigs in Uganda will be affected by heat stress in the future. The results can feed into evidence-based policy, planning and targeted resource allocation in the livestock sector.

## Implications

The study highlights how spatial analysis can be a valuable tool for mapping areas where livestock species are at risk of exposure to heat stress. This is important information as countries prepare for impending impacts of climate change to inform agricultural extension and policy.

## Introduction

The livestock sector contributes to the economic and social well-being of more than a billion people across the world and remains a crucial source of income for smallholders in the coming decades (Thornton *et al.*, 2006). Animal-sourced foods are key sources of nutrients; they provide 18% and 40% of the global energy intake and protein consumption, respectively (ILRI, 2019). The global demand for livestock products is expected to double by 2050 (Rojas-Downing *et al.*, 2017). In developing and emerging economies, the sector is rapidly evolving due to the increasing demand for livestock products (Thornton, 2010; FAO, 2017).

Climate change is among the most discussed issues in the 21st century (Cooper *et al.*, 2008). The Fifth Assessment Report of the Intergovernmental Panel on Climate Change (**IPCC**) found beyond a reasonable doubt that the Earth’s climate is warming. The global average surface temperature has increased over the 20th century by about 0.6°C (IPCC, 2014a). Further, the report advises that we should expect extreme events to become more frequent and more intense as the climate changes. The potential impacts of climate change include changes in water availability (Thornton *et al.*, 2009; Nardone *et al.*, 2010), changes in quantity and quality of feeds (Chapman *et al.*, 2012), disease prevalence for livestock (Nardone *et al.*, 2010) and reduced production (Henry *et al.*, 2012) among others. The combined changes in temperature and increased frequency of heat waves cause heat stress (Rojas-Downing *et al.*, 2017; Lacetera, 2019).

Pigs are especially sensitive to heat stress as they do not have functional sweat glands and have small lungs that reduce their ability to disseminate heat by panting (D’Allaire *et al.*, 1996). Heat stress in pig increases respiration rate, negatively affects voluntary feed intake, changes feeding patterns and results in lowered reproductive performance and growth. Moreover, new genetic lines of pigs produce nearly 20% more heat than the early 1980s breeds (Brown-Brandl *et al.*, 2003). Heat stress results in a higher rate of secondary bacterial infections due to a compromised intestinal defense mechanism (Pearce *et al.*, 2013). However, studies have demonstrated that adaptation mechanisms increase pig’s resilience, survival rates and production. There is need to implement strategies such as cooling their environment (Huynh *et al.*, 2006), adjusting voluntary feed intake (Quiniou *et al.*, 2000) and selecting genetic lines that are tolerant to heat stress (Cross *et al.*, 2018) to ensure sustainable pig production under a changing climate.

The transition of current smallholder pig production systems that are not market focused toward market-oriented models, often involving the use of exotic breeds, may come at trade-offs concerning heat stress under current and future climate. With climate change and its expected negative impacts, adaptation and increasing the resilience of pig production systems should thus be a priority. This paper demonstrates an approach for mapping areas where pigs are at risk of exposure to heat stress currently, and in the future under different climate scenarios. As a proof of concept, we applied the method to Uganda, where we also quantified the total area exposed to heat stress for both current and future periods, and finally estimated the number of pigs that are at risk of exposure to heat stress. In this study, we aim to conduct a heat stress risk analysis and as such, we did not take into consideration management practices or adaptation options that might already be in place at the farm level.

## Material and methods

### Data

We used daily time step data for maximum temperature and average relative humidity from aWhere, a complete global weather dataset at a spatial resolution of 9 km (https://developer.awhere.com/api/about-our-data/weather-data) for the month of January, 2010 and tested the reliability of using monthly means from WorldClim database (Hijmans *et al.*, 2005) and CliMond (Kriticos *et al.*, 2012), as a proxy for daily means in quantifying heat stress exposure. There were no data available to test the accuracy of future global circulation models (**GCMs**). However, we note that the GCMs were downscaled using WorldClim Version 1.4 dataset as the baseline current climate. Using ESRI ArcGIS version 10.5, we randomly selected three control points, each in high pig density areas in the cool/sub-humid, cool/humid and cool/semiarid agro-ecological zones, and calculated heat stress index (**HSI**) for each day in the month and further counted the number of days agreeing with the monthly prediction and reported this as percent agreement.

We map current pig heat stress risk using long-term (1950 to 2000) monthly means of maximum temperature from the WorldClim database (Hijmans *et al.* 2005; for more information: http://www.worldclim.org) and average relative humidity, a calculated variable using relative humidity measured at 0900 and 1500 h from CliMond (Kriticos *et al.*, 2012; for more information: https://www.climond.org/). We map future pig heat stress risk by 2050s (2040 to 2069) using pre-processed future climate data acquired from the CGIAR Research Program on Climate Change, Agriculture, and Food Security (http://www.ccafs-climate.org). They include output from 18 independent GCMs (Table 1), that is, the climate projections from the Coupled Model Intercomparison Project Phase 5 upon which the recent Fifth Assessment Report (**AR5**) of the IPCC is based (IPCC, 2014b). We selected the models based on three criteria: (i) monthly averages of daily maximum temperature were available; (ii) models have spatial resolution of fewer than 10 min (~18.5 km) over Uganda and (iii) models represent basic aspects of the observed climate compared with other GCMs at a regional scale. For each GCM model, two representative concentration pathways (**RCPs**) were incorporated. Representative concentration pathways are greenhouse gas concentration trajectories adopted by the IPCC for its AR5; in this study, we used RCP 4.5 and RCP 8.5, a moderate and aggressive scenario, respectively. The datasets were already downscaled and bias-corrected using the delta method and the WorldClim Version 1.4 dataset as the baseline current climate (Ramirez-Villegas and Jarvis, 2010). We used the same relative humidity dataset to map current and future heat stress because climate model interpretations of future and past climates have assumed that relative humidity will be constant over time regardless of how the climate changes (Allen and Ingram, 2002); this assumption holds only in the low latitudes (Ingram, 2002).

**Table 1:** List of global circulation models (GCMs) used for mapping future heat stress risk in growing-finishing pig breed

GCM^1^	Resolution^2^	Source^3^
bcc_csm1_1	64 × 128	Beijing Climate Center, China Meteorological Administration, China
bcc_csm1_1_m	160 × 320	Beijing Climate Center, China Meteorological Administration, China
cesm1_cam5	130 × 130	National Center for Atmospheric Research, USA
csiro_mk3_6_0	96 × 192	Commonwealth Scientific and Industrial Research Organisation
fio_esm	64 × 128	The First Institute of Oceanography, SOA, China
gfdl_cm3	90 × 144	Geophysical Fluid Dynamics Laboratory, USA
gfdl_esm2g	90 × 144	Geophysical Fluid Dynamics Laboratory, USA
gfdl_esm2m	90 × 144	Geophysical Fluid Dynamics Laboratory, USA
giss_e2_r	90 × 144	Goddard Institute for Space Studies (NASA), USA
ipsl_cm5a_lr	96 × 96	Institut Pierre-Simon Laplace
miroc_esm	64 × 128	Japan Agency for Marine-Earth Science and Technology, Atmosphere and Ocean Research Institute (The University of Tokyo), Japan
miroc_esm_chem	64 × 128	Japan Agency for Marine-Earth Science and Technology, Atmosphere and Ocean Research Institute (The University of Tokyo), Japan
miroc_miroc5	128 × 256	Japan Agency for Marine-Earth Science and Technology, Atmosphere and Ocean Research Institute (The University of Tokyo), Japan
mohc_hadgem2_es	145 × 192	Met Office Hadley Centre, UK
mri_cgcm3	160 × 320	Meteorological Research Institute, Japan
ncar_ccsm4	192 × 288	National Center for Atmospheric Research (NCAR), USA
ncc_noresm1_m	96 × 144	Norwegian Climate Centre, Norway
nimr_hadgem2_ao	192 × 145	National Institute of Meteorological Research/Korea Meteorological Administration

1 Numerical model representing physical processes in the atmosphere, ocean, cryosphere and land surface.

2 Model resolution units in kilometres.

3 Maintainer of GCM.

### Mapping heat stress risk

**Figure 1:**
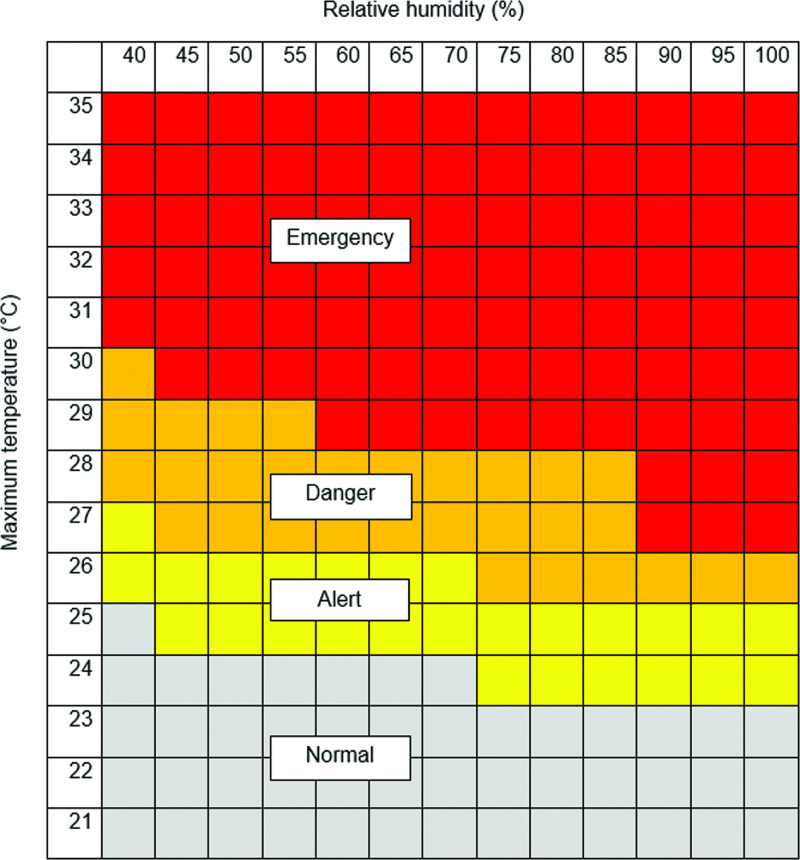
Heat stress index (HSI) for growing-finishing pig breed. Adapted from Xin and Harmon (1998) in the public domain.

The mapping was implemented in R software version 3.4.3 ‘Kite-Eating Tree’, using the following packages: rgdal version 1.4 and raster version 3.0. We mapped a HSI for pigs that combines the effects of both temperature and relative humidity and allows for the classification into four categories, that is, none, alert, danger and emergency heat stress zones (Xin and Harmon, 1998). We used this index since there was no existing equation developed for different pig breeds and climate for Uganda. Temperature and relative humidity thresholds shown in Figure 1 were used to map heat stress risk at a monthly time step for both current and future climate conditions.

We mapped future heat stress risk using 144 monthly projections (18 GCMs × 2 RCPs × 1 Period, i.e. the 2050s) and calculated the modal value for each pixel per RCP and period for constructing modal raster; the results were presented as ensemble predictions.

### Model agreement

When using GCMs in climate impacts studies, some of the sources of uncertainty associated with GCMs include length of instrumental records, emission scenario, GCM model structure and GCM downscaling method. As such, we acknowledge the uncertainty brought about by the differences between the GCMs used for the modeling, and thus we use an ensemble of models (Table 1) and calculate the number and percentage of models agreeing on heat stress categories at a given pixel per RCP and period.

### Estimation of area and number of pigs exposed

Further, we calculated the area exposed to heat stress for both current and future periods across the year and the likely changes in heat stress by identifying the difference between the two periods, that is, current and future; the result shows 16 class transitions.

Finally, to estimate the number of pigs exposed to specific heat stress categories, we overlaid the current and future heat stress maps with a pig density map (Robinson *et al.*, 2014) and calculated the number of pigs affected by different categories of heat stress across the year.

### Pig production systems in Uganda

As a proof of concept, we applied the method to Uganda where the demand for livestock products is increasing due to increasing population and income levels (Tatwangire, 2013). The livestock sector contributed about 3.5% to the total national gross domestic product as of 2019 (UBOS, 2019) and is a source of livelihood to about 58% of the population (FAO, 2019). Pig production is an important activity in Uganda. According to recent FAO statistics, it is second to beef in terms of meat production (FAO, 2018). As of 2017, Uganda had approximately 4.2 million pigs (UBOS, 2019). In the year 2013, the country had the highest per capita consumption of pork in East Africa estimated at 3.4 kg per person per year (FAO, 2018). In addition, there are expectations of an intensifying pig sector in Uganda with farmers faster embracing exotic and cross breeds such as Landrace and Large White (Tatwangire, 2013). Pig production systems in Uganda range from smallholder low-input systems, which dominate, to intensive systems, which are currently few, though increasing in number over the years.

## Results

The reliability of using monthly data as a proxy to assess heat stress exposure for pigs was investigated, and the results are presented as the percentage of days in the month of January 2010 that the daily dataset predicted the same HSI as the monthly dataset. The accuracy was on average 91.3% and, more specifically, 80.6%, 100% and 93.5% for cool/sub-humid, cool/humid and cool/semiarid agro-ecological zones, respectively.

**Figure 2:**
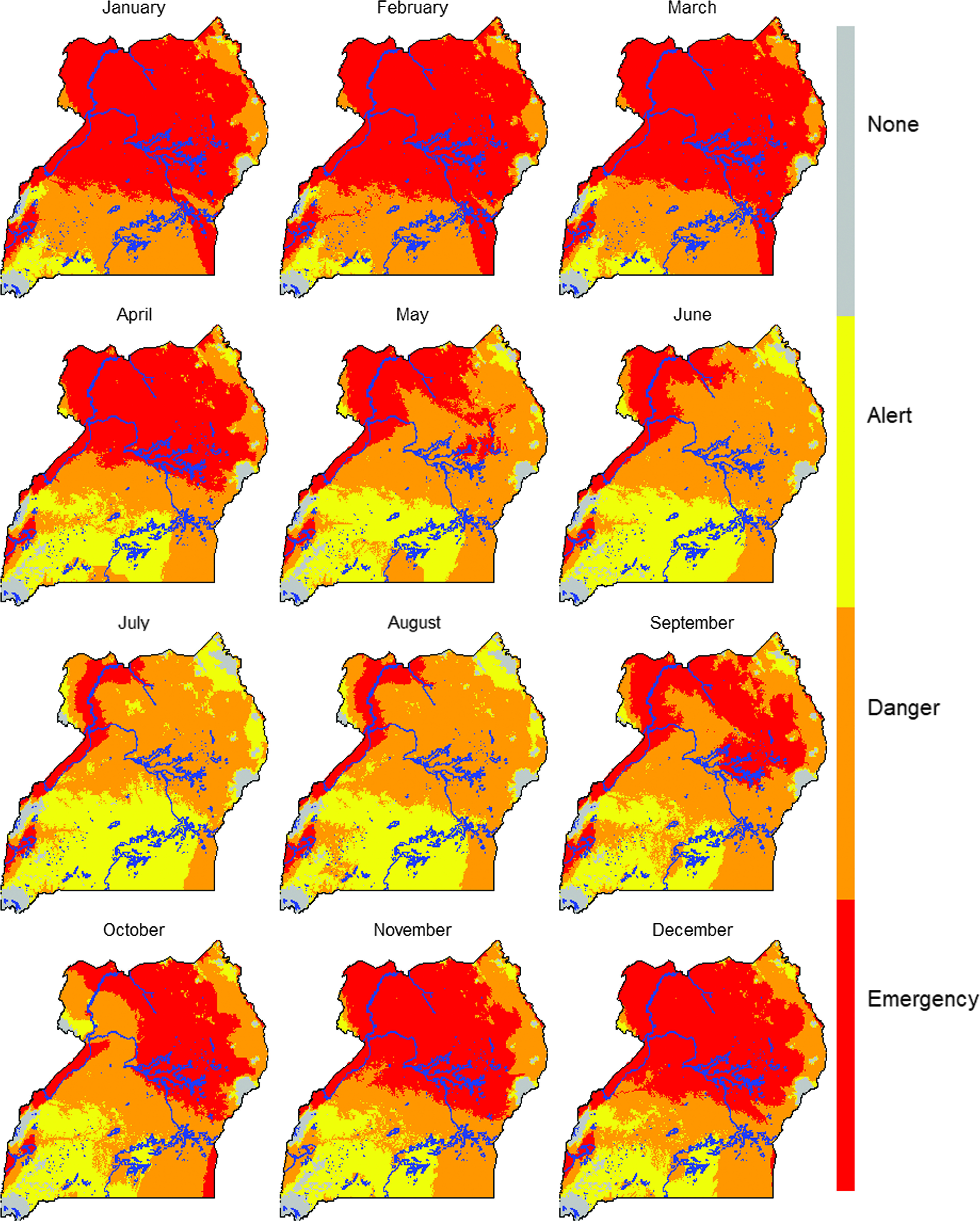
Projected areas with exposure to or at risk to heat stress for current conditions representative of the 1960 to 1990 period for growing-finishing pig breed in Uganda. Blue color indicates water bodies.

**Figure 3:**
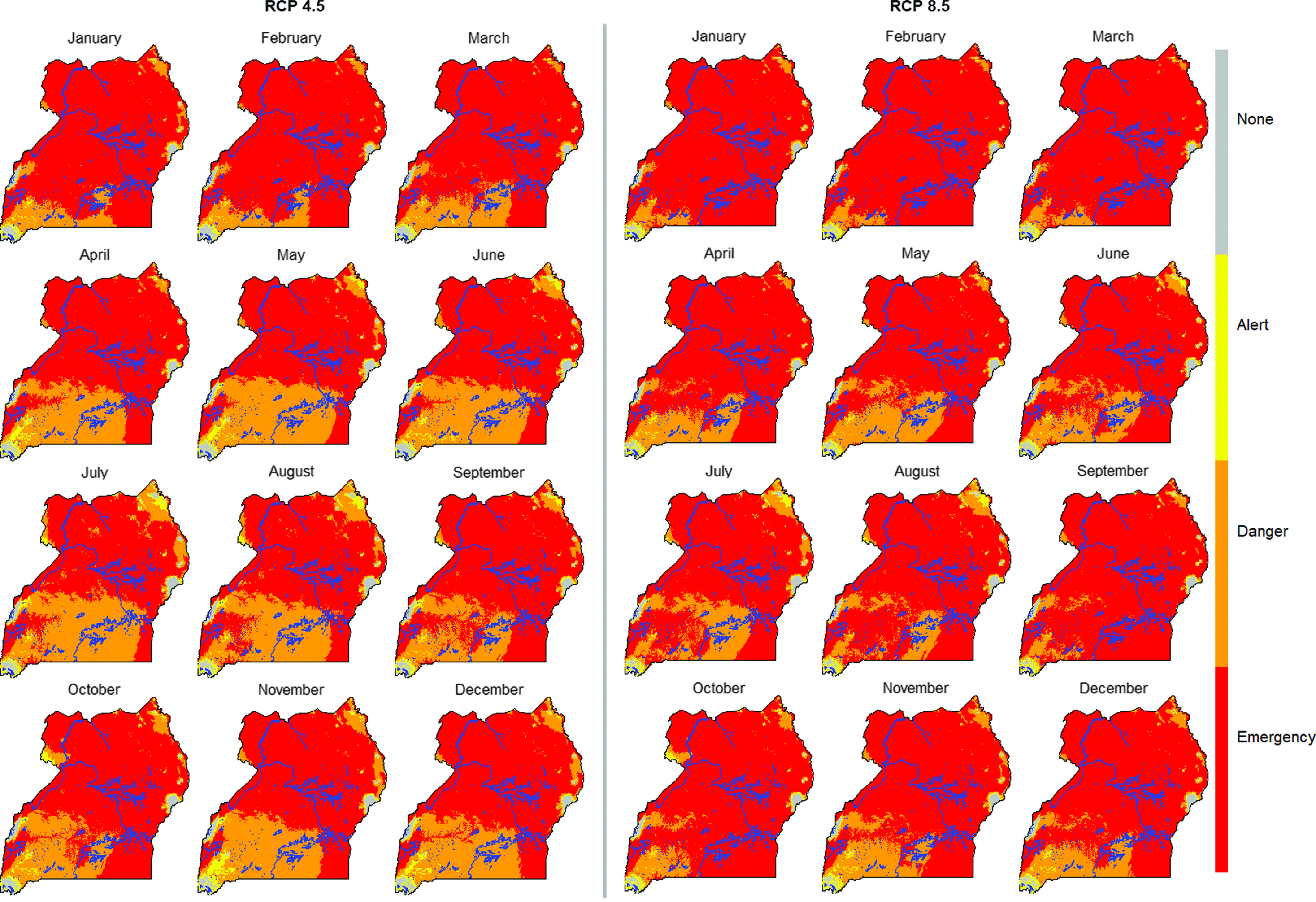
Projected areas with exposure to or at risk to heat stress for future conditions representative of 2040 to 2069 period (2050s; representative concentration pathways (RCPs): 4.5 and 8.5) for growing-finishing pig breed in Uganda. Blue color indicates water bodies.

**Figure 4:**
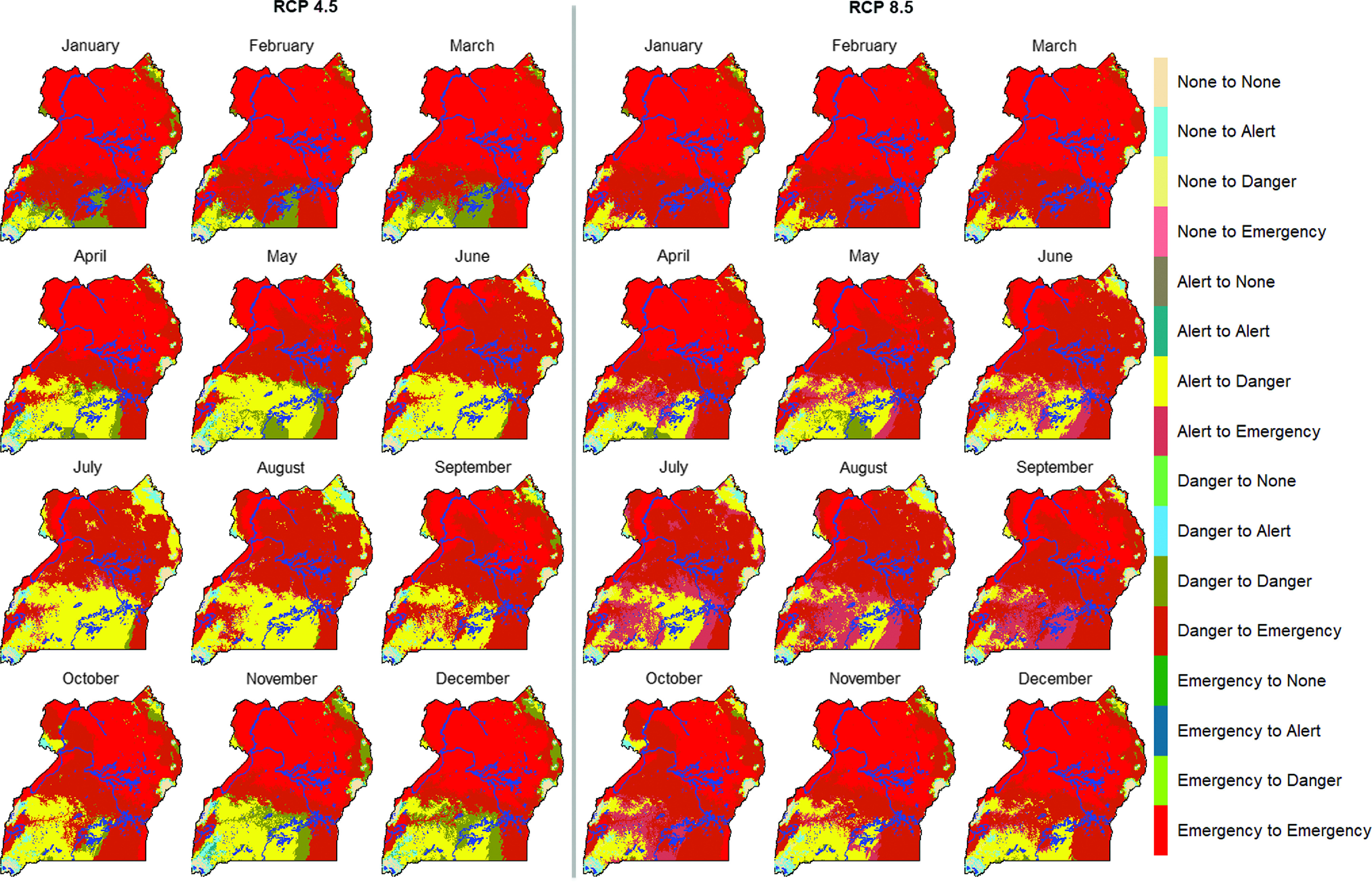
Predicted changes in exposure to heat stress with future conditions representative of 2040 to 2069 period (2050s; representative concentration pathways (RCPs): 4.5 and 8.5) for growing-finishing pig breed in Uganda. Blue color indicates water bodies.

The HSI maps suggest that most of the northern Uganda region already experiences heat stress especially during the months of October to April (Figure 2). Heat stress risk is already high and likely to increase further in the future (Figure 3). The impact of climate change is evident in the future with both emission scenarios that is, RCP 4.5 and 8.5 projecting more areas to move from danger to emergency, alert to emergency, alert to danger, none to danger and none to alert heat stress zones across the year (Figure 4). More results for future climate are reported by Mutua *et al.* (2018).

**Figure 5:**
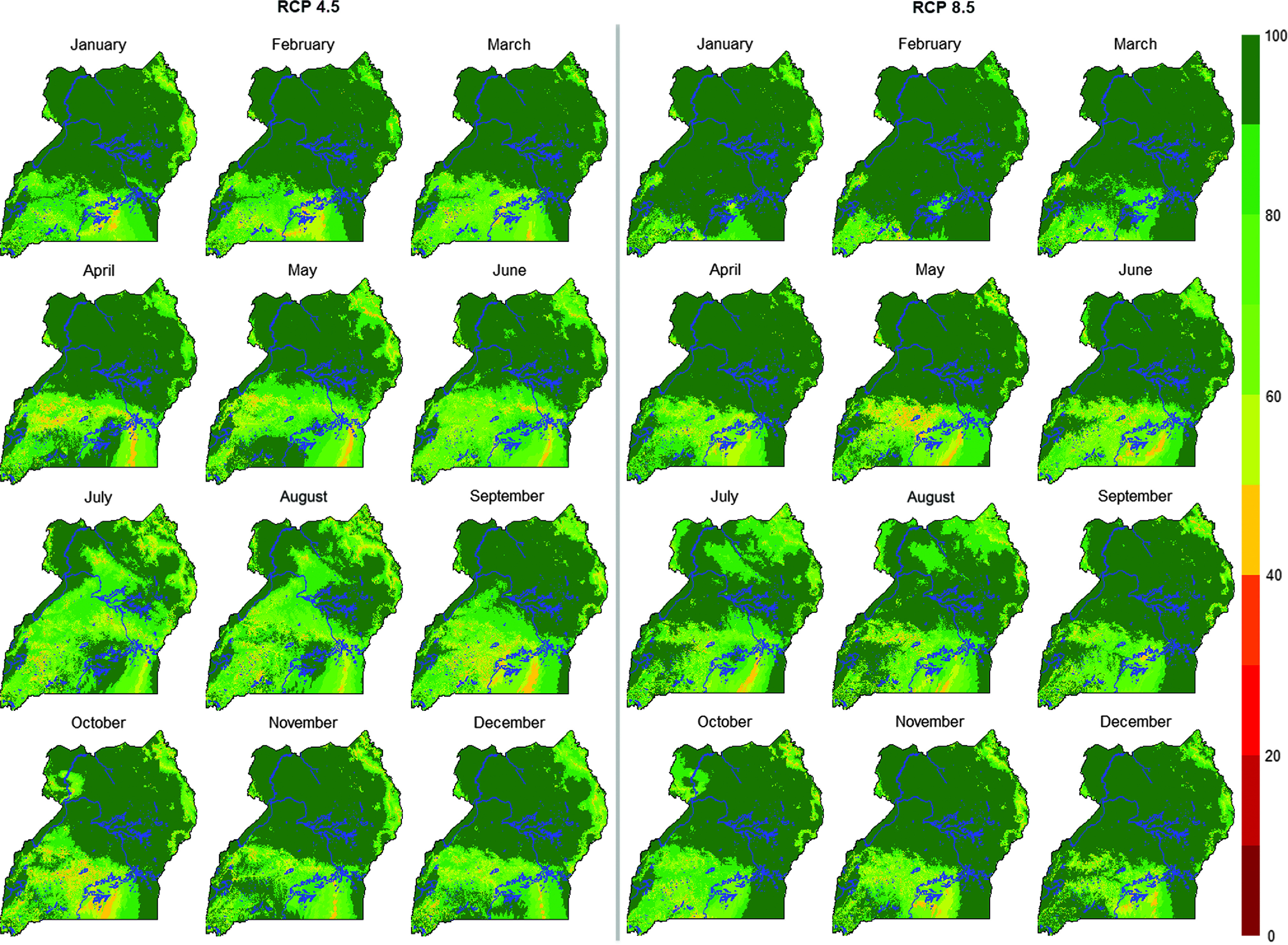
Uncertainty of future predictions in exposure to heat stress for 2040 to 2069 period (2050s; representative concentration pathways (RCPs): 4.5 and 8.5) for growing-finishing pig breed in Uganda. Blue color indicates water bodies.

Model agreement as presented in Figure 5 shows that the majority of areas within the study region have rates of agreement between models of 50% and above. Predictions for RCP 8.5 have higher confidence than predictions for RCP 4.5. In spatial terms, a lower confidence model agreement of below 50% is evident in some parts of southern Uganda, especially in the colder season.

**Figure 6:**
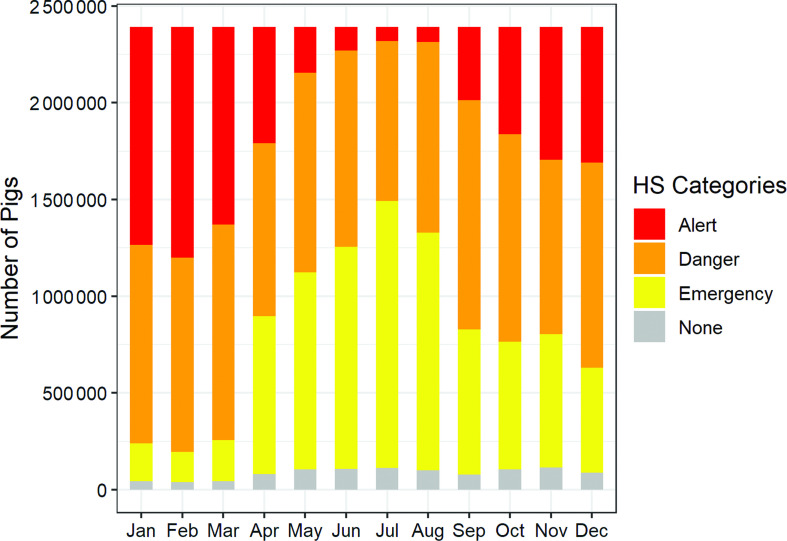
Number of pigs exposed to heat stress (HS) with current conditions representative of the 1960 to 1990 period for growing-finishing pig breed in Uganda.

Under the current climate, more than 95% of the total pig population is estimated to fall either in the alert, danger or emergency categories. The likelihood of exposure to heat stress is high in the months of January, February and March, with more than 1 million pigs estimated to be in the emergency category (Figure 6). Moreover, approximately 1 million pigs are in the danger category in the months of January, February, March, May, June, September, October and December. An additional more than 1 million pigs are in the alert category in the months of May, June, July and August. Currently, 5% of the total pig population is not exposed to heat stress across the year.

Heat stress is projected to increase in the future under all RCPs and periods with more areas exposed to heat stress (Table 2). The situation will not change for half a million pigs currently in the emergency category for both RCPs. Projections for RCPs 4.5 and 8.5 are that approximately 800 000 and 1 000 000 pigs, respectively, will move from danger to emergency category. The situation is even worse for approximately 35 000 and 300 000 pigs which are projected to move from alert to emergency category as projected under RCPs 4.5 and 8.5, respectively.

**Table 2:** Percent area under heat stress for growing-finishing pig breed in current and future conditions across months

Month	Emergency^1^			Danger^1^			Alert^1^			None^1^		
	C^2^	R1^3^	R2^4^	C	R1	R2	C	R1	R2	C	R1	R2
1	60	85	92	32	12	6	5	1	1	2	2	1
2	63	87	93	30	10	5	4	1	1	2	1	1
3	61	82	93	31	16	5	6	1	1	2	1	1
4	44	71	82	32	26	16	21	2	1	3	2	1
5	23	63	76	48	33	21	26	2	1	4	2	1
6	14	65	78	51	31	19	30	2	2	4	2	2
7	9	59	76	47	37	21	39	3	2	5	2	2
8	9	65	83	54	30	14	32	3	2	5	2	1
9	29	76	91	47	21	6	20	2	1	4	2	1
10	34	74	87	44	22	10	18	2	2	4	2	1
11	44	67	79	32	28	18	19	3	2	4	2	1
12	48	69	81	33	27	16	16	2	2	3	2	1

Units for C, R1, R2: Percent of the total area.

1 Heat stress classification zone derived from a combination of average maximum temperature (°C) and average relative humidity (%).

2 Current conditions representative of the 1960 to 1990 period by Hijmans *et al.* (2005).

3 Future conditions representative of 2040 to 2069 period (2050s) for representative concentration pathway (RCP) 4.5.

4 Future conditions representative of 2040 to 2069 period (2050’s) for representative concentration pathway (RCP) 8.5.

## Discussion

Although pork is only second to beef in terms of meat production in Uganda, the current climate change adaptation policy has little on the pig sector. Our findings show, however, that there is a need to prepare for the impending impact of heat stress on pigs in terms of agricultural extension and policy, as heat stress levels are already high and likely to increase further in the future. This could result in negative impacts on livelihoods and economy in Uganda. The results presented in this study thus highlight how spatial analysis can be a valuable tool for identifying and mapping zones where specific livestock species are at risk of exposure to heat stress. The output can feed into evidence-based policy planning and targeted resource allocation in the livestock sector so that farmers can be guided and supported in heat stress adaptation planning.

Pigs are not the only livestock species that are vulnerable to heat stress, depending on its intensity and duration, heat stress affects other species (Belhadj *et al.*, 2015; Das *et al.*, 2016; Fodor *et al.*, 2018). Expanding the current analysis to other livestock species and different breeds is thus paramount. Some recent studies have analyzed the impact of heat stress on livestock production. For example, Fodor *et al.* (2018) using 11 GCMs conducted a temporal analysis of dairy milk production in the United Kingdom for the 21st century and predicted an annual reduction of 170 kg per cow in South East England. Although not spatially explicit, Key *et al.* (2014) used four GCMs and predicted heat stress-related milk production reduction of about 0.60% to 1.35% or between USD 2000 and USD 5000 by 2030 in the USA. Although the variables used in the present study are easily available, experiments to determine the thresholds are expensive and hardly done in the tropics.

The primary heat stress adaptation strategy has been physically modifying the pig’s environment (Mayorga *et al.*, 2019; Schauberger *et al.*, 2019). There is an opportunity for breeding heat stress-tolerant pigs (Bloemhof *et al.*, 2008) although there is limited research about different species’ and breeds’ sensitivity to heat stress and potential trade-offs between sensitivity and productivity. As such, there is a need for more research on current heat stress impacts because there lies a potential to keep breeds that are productive as well as resilient and adapted to heat stress as an adaptation measure.

Finally, we acknowledge the limitation of this study. Pig’s risk of exposure to heat stress includes not only the exceedance of defined thresholds but also the duration of the exceedance, solar radiation and diurnal temperature variation among others. We used monthly means of maximum temperature which can be low at times making the animal dissipate heat. We conducted a heat stress risk analysis and did not consider management practices that might already be in place at the farm level and that could mitigate some of the potential heat stress as discussed by Zaake (2019). Further, the thresholds used in this study were mainly formulated based on experiments conducted by Xin and Harmon (1998) for growing-finishing pigs. As such, the heat stress experienced by the different pig breeds found in the Ugandan productions systems might be less severe if pigs kept in Uganda were represented in the thresholds used.

Potential next steps include applying this novel methodology to other livestock species and/or breeds as an early warning system using observed as well as weather forecast data. There is a need for more research to quantify the effect of heat stress on animal’s body metabolism, growth as well as feed intake for tropical livestock breeds. In addition, the method can be improved and linked with livestock productivity decreases and associated economic losses for quantifying potential losses in the livestock sector and economy from heat stress.

## Software and data repository resources

Mutua *et al.* (2018), GitHub repository, https://github.com/ymutua/heat-stress-mapping, available on request.
